# Characterization and Quantification of Capsaicinoids and Phenolic Compounds in Two Types of Chili Olive Oils, Using HPLC/MS

**DOI:** 10.3390/foods11152256

**Published:** 2022-07-28

**Authors:** Tilen Zamljen, Ana Slatnar, Metka Hudina, Robert Veberic, Aljaž Medic

**Affiliations:** Department of Agronomy, Biotechnical Faculty, University of Ljubljana, SI-1000 Ljubljana, Slovenia; ana.slatnar@bf.uni-lj.si (A.S.); metka.hudina@bf.uni-lj.si (M.H.); robert.veberic@bf.uni-lj.si (R.V.); aljaz.medic@bf.uni-lj.si (A.M.)

**Keywords:** capsaicinoids, HPLC/MS, health, phenolics, secoiridoids

## Abstract

Chili olive oil is a popular addition to various foods in many countries. In a detailed study, the content of phenols and capsaicinoids in chili olive oil was determined using chili flakes and whole chilies. A total of 99.8% of the phenols in chili olive oil were secoiridoids, with elenolic acid, oleuropein aglycones, and ligostride aglycones being the most abundant. Chili olive oil with chili flakes contained higher levels of capsaicinoids (+21.6%) compared to whole chili olive oil. Capsaicin and dihydrocapsaicin accounted for about 95% of all capsaicinoids in the chili olive oil. The extraction rate of dry “Cayenne” chili was 7.1% in whole chili olive oil and 9% in chili olive oil with flakes, confirming that chili flakes are better extracted in olive oil. With the determination of 29 individual phenols and five individual capsaicinoids, the study provided a detailed insight into the secondary metabolites of chili olive oil and confirmed that it is a health source.

## 1. Introduction

The olive tree has been cultivated for more than 6000 years and has spread throughout the world from Europe to California, Chile, Argentina, South Africa, and Australia [1]. Most of the olives harvested are used to produce olive oil, of which there are several varieties that differ from each other due to the specific cultivars grown in different regions [2]. Besides well-known fatty acids, olive oil contains many other useful bioactive compounds, such as aldehydes, alcohols, esters, hydrocarbons, ketones, furans, and other compounds. Polyphenols such as hydroxytyrosol, tyrosol, caffeic acid, coumaric acid, and *p*-hydroxybenzoic acid enhance the aroma and flavor of olive oil [3]. Olive oil is classified into different quality groups, such as virgin olive oil and extra virgin olive oil. The differences between them are based on the area of cultivation, the way the oil is processed (cold pressing, hot pressing), the time of harvesting, the speed of processing, and consequently the aroma and flavor at the end of the process [4].

Chilies are a popular spice added to many dishes [5]. They are rich in primary metabolites such as sugars and organic acids, especially ascorbic acid, and secondary metabolites such as phenolics and capsaicinoids [6]. Capsaicinoids are the pungent molecules found only in the *Capsicum* genus [7]. As chilies are added as spices to sauces, chocolate, jelly, and meat, they are used in many cuisines [8]. In recent decades, a very popular trend of adding chilies as spices to olive oil has emerged, especially in North America and Europe [9]. The addition of different spices such as garlic, basil, thyme, oregano, and rosemary to olive oil is well known in the Mediterranean region, so it is not surprising that the addition of chilies to olive oil was first started in this area [10]. The addition of chilies imparts a pungent flavor to olive oil that can add value to dishes seasoned with them, since they are soluble in non-polar solvents, such as oil [11].

Capsaicinoids, in moderate amounts, have beneficial effects on human health, particularly weight loss, heart disease prevention, cancer prevention, pain relief, and lowering blood sugar levels [12]. Olive oil contains many antioxidants, monounsaturated fatty acids, and several vitamins, all of which contribute to the prevention of heart disease and chronic inflammation, and lowering blood pressure [13].

In our study, we prepared two chili olive oils, one with whole chilies and the second with chili flakes from the *Capsicum annuum* L. “Cayenne” cultivar. We tried to determine the amount of capsaicinoids extracted from the chilies into the olive oil. We also determined the phenolic profile of the virgin olive oil used. The novelty of this study is that we determined 29 individual phenols and five individual capsaicinoids in the chili olive oil, which has hardly been done before. With the results of our study, we aimed to answer the question of whether chili flakes are better extracted in olive oil than whole chilies, and to show which chili olive oil is better for consumption and thus could be more beneficial for consumers’ health.

## 2. Materials and Methods

Chilies of the cultivar *C. annuum* “Cayenne” were grown at the Biotechnical Faculty (46°3′4″ N; 14°30′18″ E) of the University of Ljubljana (Ljubljana, Slovenia) from 25 May 2021 to 25 September 2021. Ten chilies were harvested on 10 September and placed in an air-drying oven at 70 °C as reported by Mihindukulasuriya and Jayasuriya [14]. After the stems were removed, half of the dried chilies were cut into flakes and the other half were left whole. The dry flakes and whole chilies were placed in two 300 mL bottles of virgin olive oil obtained from a local producer. The weight of the added whole chiles was the same as the weight of the chili flakes (in both oils 15 g of chilies were added, whether whole chiles or flakes). Maceration was performed for 14 days in a dry, dark room as previously reported by Paduano et al. [15]. After 14 days, samples of the chili olive oil were taken from each bottle to extract the phenols and capsaicinoids.

Two treatments were established: (i) chili olive oil with flakes (flakes), and (ii) chili olive oil with whole chilies (whole). To determine the success of the extraction of chili flakes and whole chilies in olive oil, we extracted dry “Cayenne” chilies to determine the initial capsaicinoid and phenolic content. The phenolic profile of the olive oil was also established.

### 2.1. Extraction of Phenolics and Capsaicinoids from Chili Olive Oil and Dry Chilies

The extraction procedure of phenolics and capsaicinoids was based on Zamljen et al. [16], with minor modifications. One mL of chili olive oil was extracted with three mL of 70% MeOH. The dry “Cayenne” chilies (0.05 g of powder) were extracted with 4 mL of 70% MeOH. The samples were mixed in a vortex mixer (Top-Mix; Bioblock Scientific, Illkirch-Graffenstaden, France) and placed in a cooled ultrasonic bath for one hour. The samples were then centrifuged at 12,000× *g* for 10 min in a centrifuge (5810 R; Eppendorf, Hamburg, Germany). After centrifugation the supernatant was collected and filtered through 25 µm polyamide filters (Chromafil AO 45/25; Macherey-Nagel, Dueren, Germany) and then stored in vials at −20 °C.

### 2.2. HPLC—Mass Spectrometry Analysis of Individual Phenolic and Capsaicinoid Compounds

The identification of phenolics and capsaicinoids was carried out in a tandem mass spectrometry (MS/MS; LTQ XL; Thermo Scientific, Waltham, MA, USA) with heated electrospray ionization operating in negative ion mode, using the parameters as described by Medic et al. [17] and Zamljen et al. [18].

The phenolics were quantified in a UHPLC system (Vanquish; Thermo Scientific, Waltham, MA, USA) and capsaicinoids in a UHPLC-PDA Thermo Scientific Dionex UltiMate 3000 HPLC (Thermo Scientific, Waltham, MA, USA) system, combined with a TSQ Quantum Access Max quadrupole mass spectrometer (MS) (Thermo Fischer Scientific Institute, Waltham, MA, USA). For capsaicinoids and phenolics a Gemini C18 (Gemini; 150 × 4.60 mm, 3 u; Phenomenex, Torrance, CA, USA) column was used, operated at 25 °C. The parameters, mobile phases and conditioning were based on Zamljen et al. [6] and Medic et al. [17].

Individual phenolics and capsaicinoids were calculated based on their respective standards. If no standards could be obtained, the substances were calculated to similar substances and expressed as equivalents. All oleuropein aglycone and derivatives, elenolic acid, and ligstroside were calculated based on the oleuropein standard. All tyrosol derivatives were calculated based on the tyrosol standard. Homocapsaicin was calculated based on the capsaicin standard and homodihydrocapsaicin based on the dihydrocapsaicin standard.

The data of individual phenolics and capsaicinoids in chili olive oil were expressed as mg/100 mL. The data of dry “Cayenne” chili were expressed as mg/100 g dry weight (DW). The chromatographic data of individual phenolics in chili olive oil are presented in Figure S1. The chromatographic data of individual capsaicinoids are presented in Figure S2.

### 2.3. Chemicals

Several standards were used for phenolics and capsaicinoids. For individual phenolics tyrosol caffeic acid, *p*-coumaric acid, apigenin-7-glucoside, luteolin-7-glucoside, kaempferol-3-glucoside, quercetin-3-*O*-glucoside, quercetin-3-*O*-rutinoside, and oleuropein (Sigma-Aldrich Chemie GmbH, Steinheim, Germany) were used; for capsaicinoids capsaicin, dihydrocapsaicin, and nordihydrocapsaicin (Sigma-Aldrich Chemie GmbH, Steinheim, Germany) were used.

### 2.4. Statistical Analysis

The data were evaluated with the use of the R statistical environment. The data are expressed as means ± standard error (SE). For determination of significant differences between both chili olive oils individual phenolics and capsaicinoids, one-way analysis of variance (ANOVA) was used, with Tukey’s test. Statistical means at a 95% confidence level were calculated to determine the significance of the differences.

## 3. Results

### 3.1. Identification of Individual Phenolics and Capsaicinoids

In chili olive oil, we identified 29 individual phenolics and five individual capsaicinoids (Table 1). Eighteen of them were represented by secoiridoids, typically found in olive and olive-type products. We identified nine oleuropein derivatives and aglycones through the typical fragmentation ions *m*/*z* 345, 307 and 275, as previously reported by Drira et al. [19], Savarese et al. [20], and Olmo-García et al. [21]. Four tyrosol compounds were identified through the typical fragmentation of *m*/*z* 153 and 123, as previously reported by Drira et al. [19], Savarese et al. [20], and Olmo-García et al. [21]. Elenolic acid (EA) and elenolic acid glycoside were identified through the fragmentation MS^2^ ions *m*/*z* 371, and MS^3^ ions *m*/*z* 223 and 179 as reported by Fu et al. [22]. Two ligstroside aglycones were identified through the fragmentation of MS^2^ ions *m*/*z* 321, 275 and 361 and MS^3^ ions *m*/*z* 291 and 259, and one ligstroside derivative trough a typical fragmentation pattern of *m*/*z* 291 and 259 ions, as reported by Fu et al. [22].

One lignan was identified (syringaresinol) through the mass 417 ([M-H]^−^) and MS^2^ fragmentation of *m*/*z* 402, 387, 371, 181, and 166, as reported by Fu et al. [22].

Four flavones were identified. Apigenin-7-glucoside and apigenin were identified with the typical fragmentation pattern of MS^2^ *m*/*z* 311 and 269 as reported by Olmo-García et al. [21]. Luteolin-7-glucoside and luteolin were identified through the typical fragmentation pattern of MS^2^ *m*/*z* 285 as reported by Cardoso et al. [23].

Four flavonols were identified in chili olive oil. Kaempferol pentoside hexoside and kaempferol derivative were identified through the fragmentation pattern of MS^2^ *m*/*z* 285 and 284 as reported by Medic et al. [24]. Quercetin derivative and quercetin-3-*O*-rutinoside were identified through the typical fragmentation pattern of MS^2^ *m*/*z* 301, 300, and 179 as reported by Cardoso et al. [23].

Two hydroxycinnamic acids were identified. Caffeic acid hexoside and *p*-cumaric acid derivative were identified through their respective fragmentation of MS^2^ *m*/*z* 179 and 135 for caffeic acid hexoside and MS^4^ 163 and 119 for *p*-cumaric acid derivative as reported by Drira et al. [19].

The five capsaicinoids were identified based on the typical fragment MS^2^ *m*/*z* 137 (Table 2). Additionally, capsaicin, dihydrocapsaicin and nordihydrocapsaicin were identified based on the fragment MS^2^ *m*/*z* 195 and 122 and homocapsaicin and homodihydrocapsaicin on MS^2^ *m*/*z* 121, 182 and *m*/*z* 195, 151, respectively, as reported by Maokam et al. [25].

### 3.2. Olive Oil Phenolics Contents

The content of analyzed individual and total phenols is shown in Table 3. The olive oil contained secoiridoids, lignans, flavones, flavonols, and hydroxycinnamic acids. The main component of the olive oil was secoiridoids, which accounted for 99.8% of all phenols determined, whereas the other four groups accounted for only 0.2% or 8.56 mg 100 mL of olive oil. The main secoiridoids were elenolic acid, oleuropein, and ligostride derivatives. The content of tyrosols was lower, with the highest content of hydroxytyrosol (3,4-DHPEA) at 1.87 mg/100 mL of olive oil.

A comparison was made between pure olive oil, olive oil containing chili flakes, and whole chilies (Table 3). No differences were found between the two chili olive oils with respect to the individual phenols in terms of individual secoiridoids and individual lignans. Both chili olive oils contained higher total flavone, total flavonols, and total hydroxycinnamic acids compared to the pure olive oil, and were extracted from the “Cayenne” chili.

Approximately 5% to 12% of phenolics were extracted from the “Cayenne” chili into the olive oil, with the best extraction rate being for flavones and the lowest for flavonols.

### 3.3. Capsaicnoid Content in Chili Olive Oil

The content of individual and total capsaicinoids is shown in Table 4. In both chili olive oils, capsaicin and dihydrocapsaicin represented the majority of capsaicinoids. Nordihydrocapsaicin, homocapsaicin, and nordihydrocapsaicin were detected in lesser amounts. Chili olive oil with chili flakes contained statistically significantly higher capsaicin levels, at 6.76 mg/100 mL (+25.2%); higher dihydrocapsaicin levels, at 0.61 mg/100 mL (+20.2%); and higher nordihydrocapsaicin levels, at 0.07 mg/100 mL (+31.8%), compared to olive oil with whole chilies (Table 4). On the other hand, the olive oil with whole chilies had higher contents of homocapsaicin and homodihydrocapsaicin than the olive oil with chili flakes, with 0.61 mg/100 mL (+95.8%) and 0.12 mg/100 mL (+42.8%), respectively.

The total capsaicinoid content was higher in the chili flake olive oil, at 6.72 mg/100 mL (+21.6%), than in the olive oil with chili flakes. Dry “Cayenne” chili extraction was performed to determine the percentage of capsaicinoids extracted into the olive oil. In terms of total capsaicinoids, approximately 8% were extracted into the olive oil. The individual capsaicinoids were extracted differently for whole chilies and chili flakes, ranging from 10.1% to 13.5% for capsaicin, from 3.9% to 4.5% for dihydrocapsaicin, from 0.5% to 0.7% for nordihydrocapsaicin, from 5.7% to 11.6% for homocapsaicin, and from 1.5% to 1.8% for homodihydrocapsaicin (Table 4).

## 4. Discussion

Chili olive oil is a popular additive to foods because it improves flavor and is a valuable source of secondary plant metabolites that may have beneficial effects on human health. We determined 29 individual phenols and five capsaicinoids in chili olive oil. The predominant phenolics in chili olive oil were secoiridoids, which were mainly oleuropein, tyrosol, and ligostride derivatives and aglycones, which has also been reported in studies by Cicerale et al. [26] and Tuck and Hayball [27]. The other phenolic groups were lignans, flavones, flavonols, and hydroxycinnamic acids, which accounted for only 0.2% of the determined phenolic content of chili olive oil. The addition of dried “Cayenne” chili flakes or whole chilies did not change the phenolic profile of the chili olive oil. Differences were observed between the chili olive oil with flakes and whole chilies in terms of phenolic quantity and quality (Table 3). A small amount of chili phenolics was extracted into the oil, mainly flavones. One reason for this could be the low phenolic content of *Capsicum* spp., as previously reported by Zamljen et al. [18].

With the addition of chilies to olive oil, humans have attempted to add depth to the flavor of olive oil and have (un)willingly added another group of very health-promoting secondary metabolites, capsaicinoids. Flaked chili olive oil had 21.6% higher capsaicinoid content than whole chili olive oil. As reported by Lu et al. [7], more capsaicinoids can be extracted from ground or cut chilies than from whole chilies, which was similar in our case. The rationale for this is that the extraction area is larger for chili flakes and more cells are broken than for whole chilies. The extraction rate was relatively low, with the oil of the chili flakes containing 9% of the capsaicinoids of the original dry “Cayenne” chili, whereas the oil of the whole chilies contained 7.1% of the original dry “Cayenne” chili. Similar low extraction rates of capsaicinoids and phenolics were also reported by Paduano et al. [15] in sun flower oil. Both capsaicin and dihydrocapsaicin were predominant in both chili olive oils and accounted for about 95% of all capsaicinoids. Nordihydrocapsaicin, homocapsaicin, and homodihydrocapsaicin accounted for the remaining 5%. Similar results were also reported by Barbero et al. [28] in fresh chilies.

The two main groups of secondary metabolites in chili olive oil, namely, capsaicinoids and secoiridoids, have beneficial effects on human health, especially in reducing the risk of diabetes, inflammation, cancer, and obesity [29]. Regular consumption of foods containing these substances also reduces the risk of cardiovascular disease, as reported by Chopan and Littenberg [30]. When capsaicinoids from chilies are combined with a high-quality extra virgin olive oil containing high levels of secoiridoids and other health benefiting compounds, the resulting chili olive oil can greatly benefit human health.

## 5. Conclusions

In this detailed study, we used HPLC/MS to determine in detail 29 phenols and five capsaicinoids in two types of chili olive oil, one with chili flakes and one with whole chilies. We confirmed that chili flakes in olive oil are better because they contain more capsaicinoids, which is better for human consumption. Since previous studies only examined a handful of phenols in olive oil and the number of studies on chili olive oil and its secondary metabolite content is small, this study provides great insight and novelty into the very popular food of chili olive oil. Additionally, this study lays foundations for further studies regarding chili olive oils, in which more types of substances could be analyzed such as fatty acids.

## Figures and Tables

**Table 1:** Fragmentations of individual phenolics.

Compound	Rt	[M-H]^−^	MS^2^	MS^3^	MS^4^
	(min)	(*m*/*z*)	(*m*/*z*)	(*m*/*z*)	(*m*/*z*)
Hydroxytyrosol (3,4-DHPEA)	8.69	153	123 (100), 109 (3)		
Caffeic acid hexoside	11.98	341	179 (100), 135 (4)		
Kaempferol pentoside hexoside	19.95	579	447 (100), 285 (86), 284 (7)		
Quercetin-3-rutinoside	20.27	609	301 (100), 300 (21), 179 (3)		
Demethyloleuropein 1	21.86	525	**389** (100), 523 (96), 359 (53)	315 (100), 241 (59), 323 (51)	
Quercetin derivative	22.80	505	301 (100), 300 (69), 179 (3)		
Kaempferol derivative	22.99	622	490 (100), 489 (36), 285 (35), 284 (6)		
Elenolic acid (EA)	23.42	371	223 (100), 179 (72), 95 (47), 101 (31), 209 (27), 165 (24)		
Elenolic acid glycoside	24.56	403	**371** (100)	223 (100), 179 (76)	
Hydroxy-decarboxymethyl oleuropein aglycone	24.73	335	199 (100), 181 (6)		
Decarboxymethyl oleuropein aglycone	29.36	319	**181** (100), 111 (18), 153 (5)	121 (100), 153 (95), 111 (29)	
Luteolin	29.55	285	241 (100), 175 (80), 243 (78), 199 (76), 217 (75)		
Oleuropein aglycone 1	29.82	377	307 (100), 275 (64), 345 (18)		
Dehydroligstroside aglycone	29.95	359	**313** (100), 323 (79), 285 (38)	291 (100), 259 (75), 241 (18)	
Hydroxy oleuropein aglycone	30.01	393	**317** (100), 349 (36), 361 (19)	**289** (100), 181 (62), 137 (14)	153 (100), 229 (60), 245 (36), 179 (21)
Luteolin-7-glucoside	30.34	447	285 (100)	235 (100), 247 (96), 261 (50), 127 (43)	
Apigenin	30.51	269	225 (100), 149 (35), 201 (26), 151 (20)		
Syringaresinol	30.64	417	402 (100), 387 (76), 371 (24), 181 (9), 166 (7)		
Oleuropein derivative	30.85	413	**377** (100)	307 (100), 333 (64), 275 (63)	
3,4-DHPEA-EA	31.07	377	333 (100), 307 (87), 275 (57)		
Hydroxytyrosol glucoside 1	31.18	315	153 (100), 135 (63), 123 (22)		
Ligstroside aglycone	31.49	391	**321** (100), 275 (54), 361 (44)	291 (100), 259 (35)	
Hydroxytyrosol glucoside 2	31.64	315	153 (100), 135 (33), 123 (15)		
*p*-cumaric acid derivative	31.70	543	**419** (100)	**295** (100), 299 (44), 283 (11)	163 (100), 119 (33)
Ligstroside derivative	31.83	361	291 (100), 259 (65)		
Apigenin-7-glucoside	32.16	431	311 (100), 269 (87)		
Dehydrooleuropein aglycone	32.46	375	**305** (100), 259 (48), 151 (13)	139 (100), 111 (31)	
Oleuropein aglycone 2	32.54	377	345 (100), 307 (64), 275 (20), 241 (17), 197 (14), 165 (8)		
Demethyloleuropein 2	33.09	525	389 (100), 345 (49), 319 (32)		

**Table 2:** Identification of individual capsaicinoids.

Compound	Rt	[M-H]^+^	MS^2^
	(min)	(*m*/*z*)	(*m*/*z*)
Nordihydrocapsacin	14.10	294	137 (100), 122 (65), 195 (10)
Capsacin	14.26	306	137 (100), 133 (42), 195 (12)
Dihydrocapsacin	15.04	308	137 (100), 122 (46), 195 (17), 178 (7)
Homocapsaicin	15.19	320	137 (100), 121 (60), 182 (23)
Homodihydrocapsaicin	15.94	322	137 (100), 195 (59), 151 (21)

**Table 3:** Individual olive oil phenolic contents (mean ± SE) in mg/100 mL. Individual phenolics were compared among pure chili olive oil, chili olive oil with flakes, and chili olive oil with whole chilies. The “Cayenne” chili phenolic content is only for reference.

Individual Phenolic Compound	Chili Olive Oil (Flakes)	Chili Olive Oil (Whole)	Olive Oil (No Chili)	“Cayenne” Chili w/o Oil
Hydroxytyrosol (3,4-DHPEA)	2.01 ± 0.05	1.73 ± 0.09	1.87 ± 0.03	
Demethyloleureopein 1	0.60 ± 0.01	0.61 ± 0.02	0.60 ± 0.01	
Elenolic acid (EA)	364.07 ± 0.06	363.87 ± 0.08	363.97 ± 0.10	
Elenolic acid glycoside	364.03 ± 0.08	364.09 ± 0.11	364.06 ± 0.10	
Hydroxy-decarboxymethyl Oleuropein aglycone	364.02 ± 0.02	363.94 ± 0.06	364.02 ± 0.08	
Decarboxymethyl oleuropein aglycone	364.80 ± 0.10	363.10 ± 0.07	363.95 ± 0.04	
Oleuropein aglycone	365.23 ± 0.56	364.52 ± 0.23	364.88 ± 0.19	
Dehydroligastroside aglycone	368.13 ± 0.20	371.05 ± 0.75	369.59 ± 0.85	
Hydroxy oleuropein aglycone	368.30 ± 0.43	368.84 ± 0.37	368.57 ± 0.42	
Oleuropein derivative 1	384.86 ± 0.56	373.22 ± 0.69	379.04 ± 0.55	
3,4-DHPEA-GA	1.90 ± 0.27	0.92 ± 0.05	1.41 ± 0.15	
Hydroxytyrosol glucoside 1	0.74 ± 0.04	0.59 ± 0.07	0.66 ± 0.04	
Ligostride aglycone	364.12 ± 0.09	364.02 ± 0.17	364.07 ± 0.09	
Hydroxytyrosol glucoside 2	2.08 ± 0.07	1.54 ± 0.11	1.81 ± 0.07	
Ligostride derivative	364.55 ± 0.08	364.62 ± 0.07	364.58 ± 0.04	
Dehydrooleuropein aglycone	363.48 ± 0.03	363.48 ± 0.03	363.48 ± 0.03	
Oleuropein derivative 2	386.00 ± 0.75	392.39 ± 0.82	389.20 ± 0.69	
Demethyloleuropein 2	369.52 ± 0.18	373.46 ± 0.34	371.49 ± 0.22	
**Total secoiridoids**	**4797.74 ± 2.03**	**4795.99 ± 2.31**	**4797.25 ± 2.11**	
Syringaresinol	1.27 ± 0.07	0.89 ± 0.04	1.08 ± 0.04	
**Total lignans**	**1.27 ± 0.07**	**0.89 ± 0.04**	**1.08 ± 0.04**	
Apigenin-7-glucoside	0.15 ± 0.01 a **^§^**	0.14 ± 0.01 a	0.11 ± 0.01 b	0.29 ± 0.10
Apigenin	0.70 ± 0.01 a	0.33 ± 0.02 b	0.30 ± 0.03 a	1.53 ± 0.12
Luteolin-7-glucoside	3.84 ± 0.31 a	3.40 ± 0.24 b	2.62 ± 0.34 b	5.77 ± 0.30
Luteolin	2.31 ± 0.22 a	1.79 ± 0.58 b	1.05 ± 0.39 b	4.67 ± 0.19
**Total flavone**	**7.00 ± 0.12 a**	**5.66 ± 0.09 b**	**4.08 ± 0.10 b**	**12.26 ± 0.85**
Kaempferol pentoside hexoside	0.12 ± 0.01 a	0.15 ± 0.01 a	0.09 ± 0.01 b	0.95 ± 0.08
Kaempferol derivative	0.12 ± 0.00 a	0.11 ± 0.01 a	0.09 ± 0.01 b	0.89 ± 0.09
Quercetin derivative	0.08 ± 0.01 a	0.08 ± 0.01 a	0.05 ± 0.01 b	0.56 ± 0.02
Quercetin-3-rutinoside	0.16 ± 0.01 a	0.15 ± 0.01 a	0.11 ± 0.01 b	0.66 ± 0.07
**Total flavonols**	**0.48 ± 0.02 a**	**0.49 ± 0.02 a**	**0.34 ± 0.02 b**	**3.06 ± 0.29**
*p*-cumaric acid derivative	0.10 ± 0.01 a	0.11 ± 0.01 a	0.08 ± 0.01 b	0.47 ± 0.06
Caffeic acid hexoside	0.41 ± 0.01 a	0.42 ± 0.01 a	0.38 ± 0.02 b	0.73 ± 0.06
**Total hydroxycinnamic acids**	**0.51 ± 0.03 a**	**0.53 ± 0.02 a**	**0.46 ± 0.02 b**	**1.20 ± 0.24**
**Total analyzed phenolics**	**4807.85 ± 2.66 a**	**4803.76 ± 2.46 a**	**4805.81 ± 2.54 a**	**16.52 ± 1.05**

^§^ a, b letters denote statistical differences among pure olive oil, chili olive oil with flakes, and chili olive oil with whole chilies. Where no statistical differences were observed, no letters were written.

**Table 4:** Individual and total capsaicinoid content (mean ± SE) in chili olive oil and dry “Cayenne” chili before being added to the olive oil.

	Chili Olive Oil—Flakes	Chili Olive Oil—Whole	“Cayenne” Chili w/o Oil
Capsaicin	26.84 ± 0.31 b **^§^**	20.08 ± 0.31 c	198.53 ± 1.34 a
Dihydrocapsaicin	3.02 ± 0.04 b	2.41 ± 0.08 c	61.79 ± 1.01 a
Nordihydrocapsaicin	0.22 ± 0.01 b	0.15 ± 0.01 c	48.69 ± 0.73 a
Homocapsaicin	0.64 ± 0.02 c	1.25 ± 0.07 b	10.77 ± 0.22 a
Homodihydrocapsaicin	0.28 ± 0.01 c	0.40 ± 0.01 b	21.25 ± 0.13 a
**Total capsaicinoids**	**31.02 ± 0.37 b**	**24.30 ± 0.28 c**	**341.03 ± 1.63 a**

^§^ different letters (a, b, c) presents statistically significant differences among both chili olive oils and the “Cayenne” chili.

## Data Availability

The data presented in this study are available on request from the corresponding author. The data are not publicly available due to privacy.

## Supplementary Materials

The following supporting information can be downloaded at: https://www.mdpi.com/article/10.3390/foods11152256/s1, Figure S1: Chromatographic data of phenolics in chili olive oil from retention time of 5 min to 28 min (Figure S1A) and from 28 min to 36 min (Figure S1B); Figure S2: Chromatographic data of capsaicinoids in chili olive oil.
