# Supplementary material for: Characterization and Quantification of Capsaicinoids and Phenolic Compounds in Two Types of Chili Olive Oils, Using HPLC/MS
Source: Foods. 2022 Jul 28;11(15):2256. doi: 10.3390/foods11152256 (PMC9367771; doi:10.3390/foods11152256)
Supplement: Supplementary file 1 [file foods-11-02256-s001.zip › foods-1831748-supplementary.pdf]

Figure S1: Chromatographic data of phenolics in chili olive oil from retention time 5 min to 28 min (Figure **A**) and from 28 min to 36 min (Figure **B**).

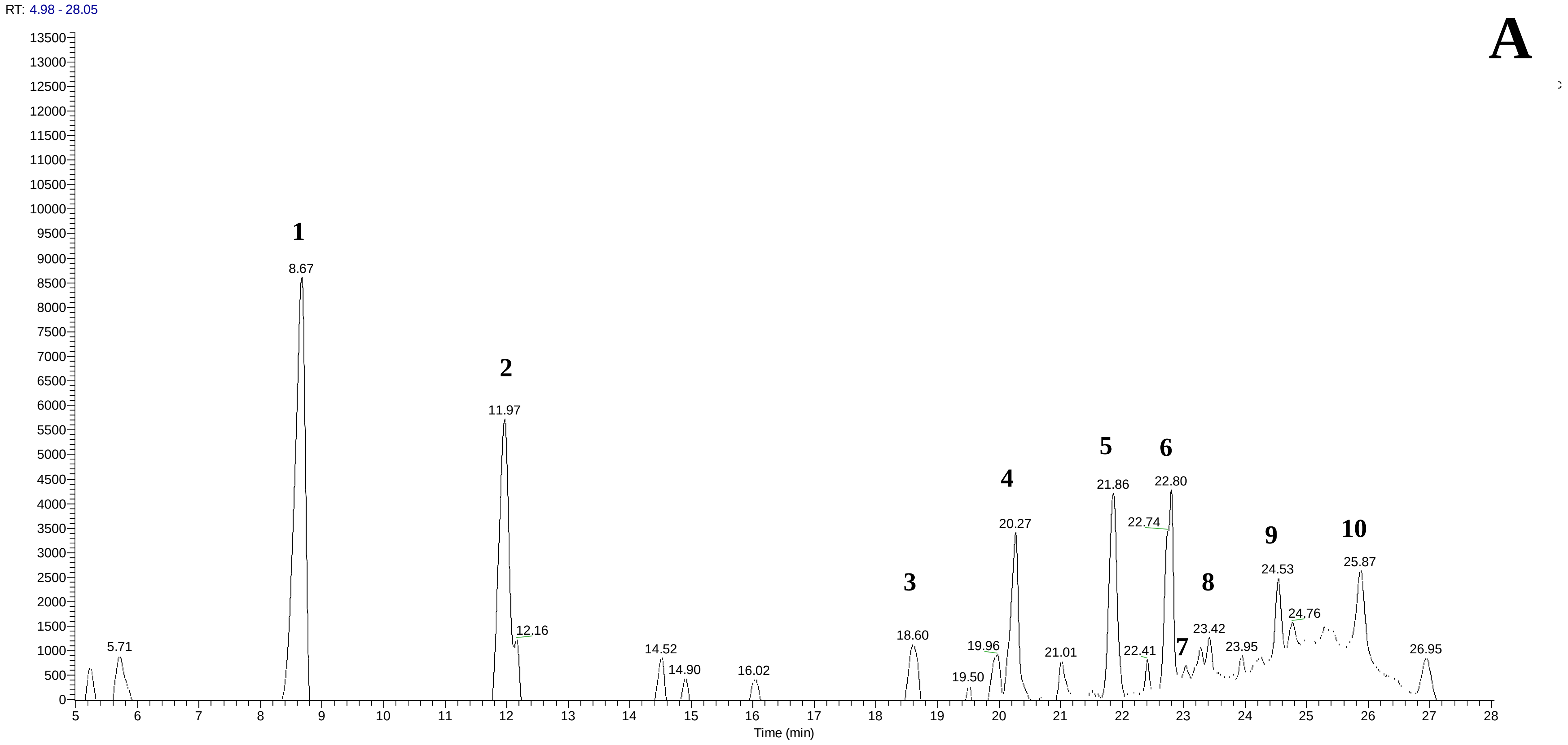

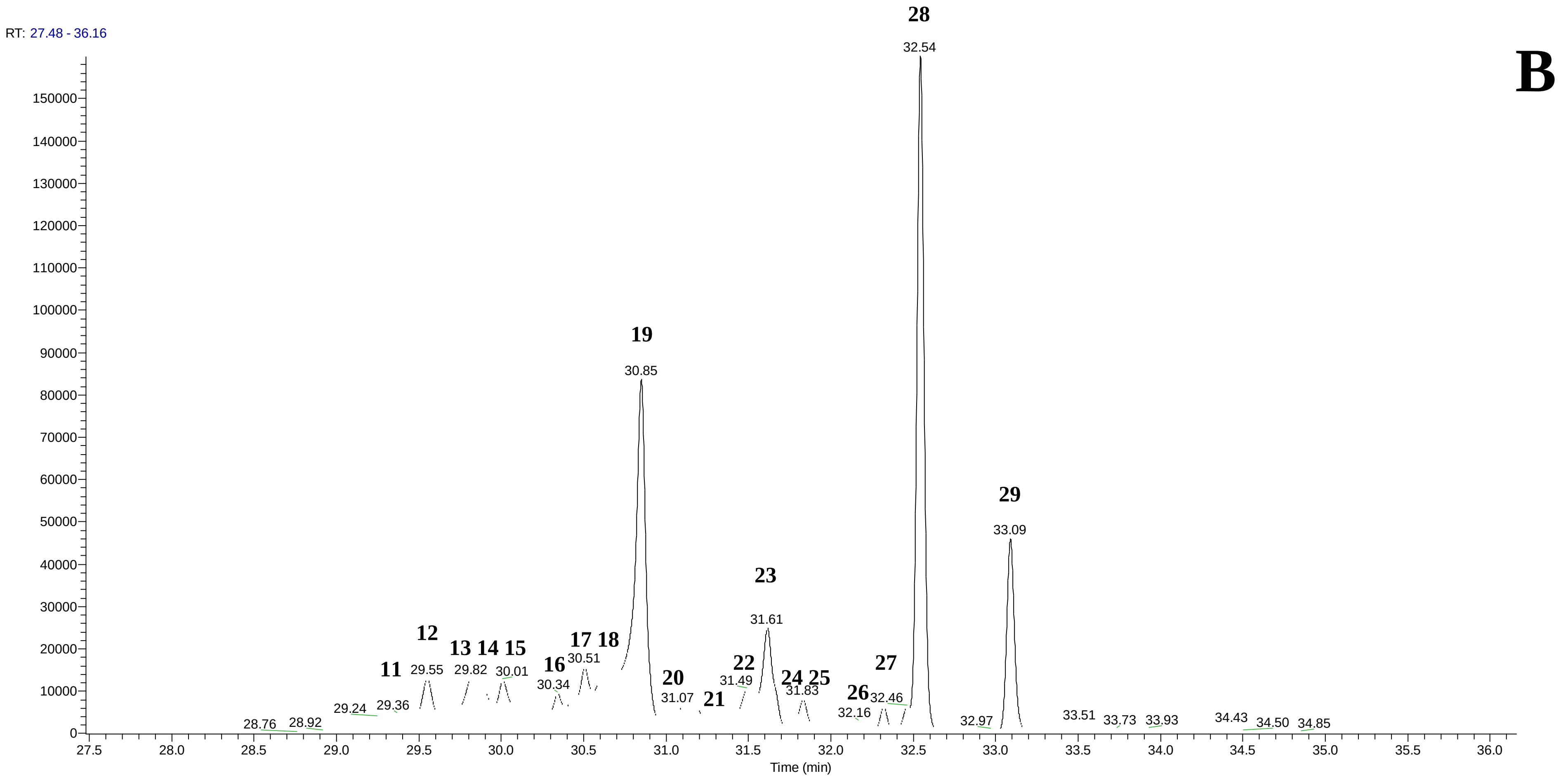

1= hydroxytyrosol (3,4- DHPEA); **2**= caffeic acid hexoside; **3**= kaempferol pentoside hexoside; **4**= quercetin-3-*O*-rutinoside; **5**= demethyloleuropein 1; **6=** quercetin derivative; **7**= kaempferol derivative; **8**= elenolic acid (EA); **9**= elenolic acid glycoside; **10**= hydroxy-decarboxymethyl oleuropein aglycone; **11**= decarboxymethyl oleuropein aglycone; **12**= luteolin; **13**= oleuropein aglycone; **14**= dehydroligstroside aglycone; **15**= hydroxy oleuropein aglycone; **16**= luteolin-7-glucoside; **17**= apigenin; **18**= syringaresinol; **19**= oleuropein derivative 1; **20**= 3,4- DHPEA-EA; **21**= hydroxytyrosol glucoside 1; **22**= ligstroside aglycone; **23**= hydroxytyrosol glucoside 2; **24=** *p*-cumaric acid derivative; **25**= ligstroside derivative; **26**= apigenin-7- glucoside; **27**= dehydrooleuropein aglycone; **28**= oleuropein derivative 2; **29**= demethyloleuropein 2

Figure S2: Chromatographic data of capsaicinoids in chili olive oil.

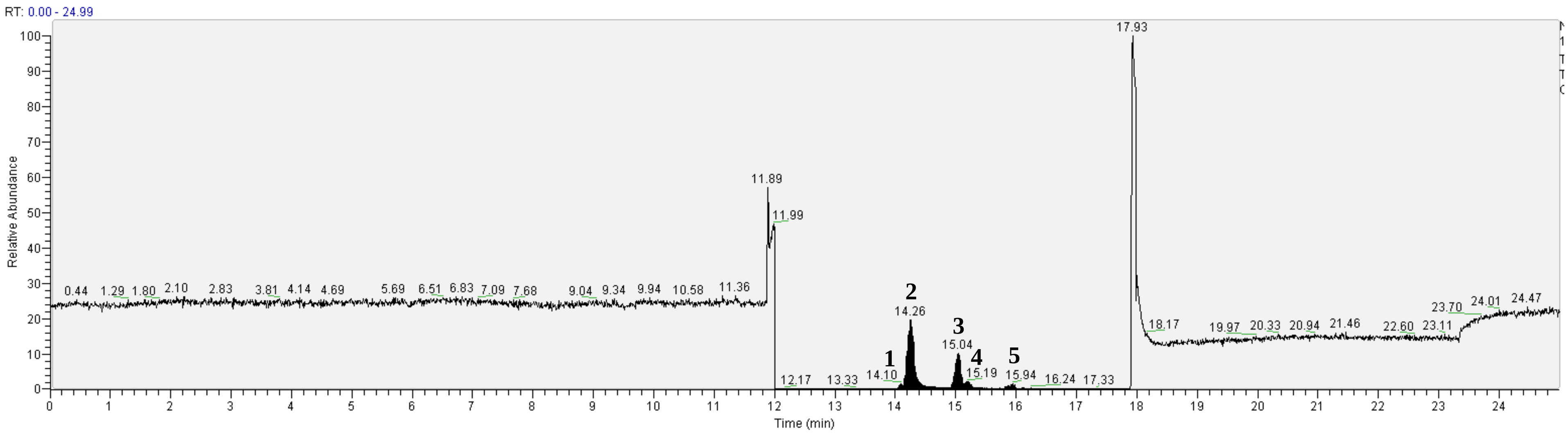

1= Nordihydrocapsaicin; **2**= Capsaicin; **3**= Dihydrocapsaicin; **4**= Homocapsaicin **5**= Homodihydrocapsaicin
